# Partially ionized plasma of the solar atmosphere: recent advances and future pathways

**DOI:** 10.1098/rsta.2023.0230

**Published:** 2024-06-09

**Authors:** Andrew S. Hillier, Ben Snow, Manuel Luna

**Affiliations:** ^1^ Department of Mathematics and Statistics, University of Exeter, Exeter EX4 4QF, UK; ^2^ Departament de Física and Institute of Applied Computing & Community Code (IAC^3^). Universitat de les Illes Balears, 07122 Palma de Mallorca (Spain)

In many ways, understanding the energy flow and dynamic motions in the solar atmosphere is the key to revealing many of the mysteries about the Sun, including how the atmosphere of the Sun is heating and the formation of cool clouds in the solar atmosphere called prominences. Throughout the solar atmosphere magnetic fields are of fundamental importance for the energy transport and in driving a wide range of dynamics. However, in the lower layers of the atmosphere, the fluid in which they are embedded is predominantly neutral. This means that the majority of the fluid is not directly affected by the forces of the magnetic field. For this type of fluid, known as a partially ionized plasma, it is interactions between the neutral particles and the charged ions and electrons that allow the magnetic field to couple with the neutrals and drive coherent dynamics. This process, which takes a finite time, allows neutral material to drift across the magnetic field and leads to dissipation of magnetic energy, which results in heating of the plasma. Ultimately, this can have significant consequences for our understanding of dynamics and energy flow.

The processes that control the coupling between the two fluids happen at small scales and high frequencies, as such historically these processes have not been accessible to observations, making direct study of the effects of partial ionization in the solar atmosphere difficult. However, we have recently entered a new age of solar observations; with the first light of the Daniel K. Inouye Solar Telescope (DKIST), we now have a ground-breaking new instrument (the first 4 m solar telescope) that allows us to study the Sun in unprecedented detail. Combined with other world-class telescopes like the New Vacuum Solar Telescope, upcoming missions like NASA’s Multi-slit Explorer (MUSE) and the proposed 4 m European Solar Telescope, we will soon have a huge amount of observational data that will shine a light on the small-scale, high-frequency dynamics that are seen as crucial for heating in the solar atmosphere. A key science objective of these missions is to observe the lower solar atmosphere where the plasma is partially ionized giving them great potential to bring new insights into the physics of the partially ionized solar atmosphere.

In addition to the revolutionary observational advances that will be provided by these new instruments, there have been substantial, recent advances in the theoretical modelling that have come about through novel treatment of underlying physics. Until recent years aspects of the solar atmosphere such as the radiative transfer, which determines the energy transfer and the ionization of the medium, and the multi-fluid interactions have been beyond the scope of models. New studies which include new and improved modelling of the previously mentioned physical processes are pushing the boundaries of our understanding. By building more realistic models, this is leading to greater depth of knowledge of partially ionized plasma dynamics and the structures of the solar atmosphere that host them.

The observations and modelling of partially ionized plasma will form a key part of the development in this new frontier of solar physics. As a consequence, over the last 10 years, there has been increasing interest in the cool material of the solar atmosphere, in particular, how the magnetic fields that drive dynamics and heating of this material can interact with neutral species. This has resulted in a number of groups developing their own studies of partially ionized plasma in the solar atmosphere, and we have likely reached a tipping point as two-fluid and multi-fluid modelling moves towards becoming the standard for studying partially ionized plasma.

Despite the growth in interest in partial ionization, the field is still in its infancy. The underlying physical processes that drive and control partially ionized plasmas are not well understood and are mostly studied in isolation. This includes researchers who are advancing the field through their work on MHD theory, plasma physics, numerical modelling, atomic processes and solar observations. A unified understanding of all these processes is required to comprehend the true nature of the lower solar atmosphere with various efforts (including this thematic issue) coming from the community.

One such effort to tackle the big questions in partial ionization of the solar atmosphere was led by Prof. Jose-Luis Ballester and Dr Manuel Luna who organized for a research team to be hosted at the International Space Science Institute (ISSI) in Bern, Switzerland, for two discussion meetings. The focus of these meetings was ‘The Role of Partial Ionization in the Formation, Dynamics and Stability of Solar Prominences’. Solar prominences are fascinating structures embedded in the solar corona. Their peculiar properties and behaviour, not yet well understood, cause them to be the subject of intense research. Furthermore, they are an integral part of major solar eruptions, therefore, greater understanding of their formation and evolution will contribute significantly to our understanding of the origin of space weather, a serious threat to our technology-dependent world. Due to their relatively low-temperature prominence plasma is partially ionized, but the exact ionization degree is unknown and the reported ratio of electron density to neutral hydrogen density covers about two orders of magnitude (0.1–10). This makes prominences an interesting and important focus for research in partially ionized plasma.

There were a number of key takeaways from these two meetings. Models for the radiative transfer (which determines the ionization degree of the plasma) and of the dynamics of the plasma itself have reached a level of maturity where the next step is to work to bring them together. We learnt that there are some very creative ideas under development and approaches now being used in the field (with many of them being presented in this special issue). Finally, it is now clear that our theoretical understanding of solar partially ionized plasma has now reached the point where we can make direct predictions that can be tested by observations, and have the observational instrumentation to actually test those predictions. Many of the scientific advances presented, discussed and developed during this meeting have become papers for this theme issue.

This theme issue of *Philosophical Transactions of the Royal Society A*, titled ‘Partially ionized plasma of the solar atmosphere: recent advances and future pathways’, aims to present the cutting edge of the current research on the partially ionized sun, as well as providing a roadmap for future developments. We do this by both capturing the research outputs from the ISSI meetings as well as bringing in other studies from different groups from around the world to showcase the recent developments in the understanding of solar partially ionized plasma.

The first papers of this issue look at some of the underlying physics of partial ionized plasma modelling, both from a radiative transfer perspective and from a dynamics perspective. Heinzel & Gunar [1] present a review of the physics behind the ionization of prominence material where they demonstrate that for a typical prominence, it is photoionization that is the dominant contribution to the partial ionization and this mechanism strongly depends on how the radiation field of the surrounding plasma illuminates the prominence. The single-fluid approximation for partially ionized plasma (involving the addition of the ambipolar diffusion term to the induction equation) is a regularly used modelling tool. Gómez Míguez *et al*. [2] made a detailed comparison between this approximation for partially ionized plasma modelling and the more detailed two-fluid model (solving for both the dynamics of neutrals and charges), highlighting the importance of the extra force terms that are not readily captured in single-fluid approximations. Taking a different perspective, Hillier [3] looked at how to relax one of the underlying assumptions behind the development of single-fluid partially ionized plasma modelling showing that when the velocity difference between charges and neutrals gets large enough this has a negative feedback on the rate at which neutrals drift across the magnetic field.

MHD waves are of particular importance in solar physics due to the ability of waves to transport energy that can then be dissipated to heat the upper atmosphere. As these waves are ubiquitous in the partially ionized solar atmosphere, understanding the influence of partial ionization on MHD waves is a key topic of this issue. To lead this area, the review article by Soler [4] provides a comprehensive overview of the current understanding of the field. Ballester *et al.* [5] studied the coupling of nonlinear Alfvén waves to thermodynamics in partially ionized plasma finding a complex interplay of damping mechanisms for the waves. Looking at the coupling of Alfvén waves to ionization-recombination waves, Ballai *et al.* [6] found that the Alfvén waves could be parametrically excited by periodic changes in the ionization degree. Through two-fluid numerical simulations, Kuzma *et al*. [7] studied the influence of partial ionization in the lower solar atmosphere on the acoustic cut-off frequency of gravity waves, finding the partial ionized effects are necessary to match the numerical modelling to solar observations. Srivastava *et al.* [8] used two-fluid numerical simulations to model the formation of thin jets, called spicules, in the solar atmosphere with the ion-neutral effects, an important aspect of driving a high height of the simulated spicules.

Instabilities can be viewed as the other side of the coin to waves, driving large growth to perturbations to the system and the release of stored energy in the system. A critical instability for the development of solar prominences and coronal rain is the thermal instability. Popescu Braileanu & Keppens [9] analytically and numerically investigate the growth of this instability in the partially ionized plasma of the solar atmosphere showing important changes in the growth rate and dynamics from the fully ionized case. Another key aspect of instabilities is the ability to mix different material together, with the neutrals in partially ionized plasma able to act as a transport mechanism for mass and thermal energy. Snow & Hillier [10] looked at the role of the Kelvin–Helmholtz instability and Lukin *et al.* [11] looked at the Rayleigh–Taylor instability to mix prominence plasma. Both studies highlighted different dynamical processes that could drive ionization and recombination and with that complex thermodynamic evolution.

To provide a look forward in this issue, Parenti *et al.* [12] present a piece looking at the future perspectives of the field. We hope that by bringing these topics together and providing our view for future progress we can contribute to the continued growth of this research field. Particularly through inspiring a new generation of researchers to join the field.

## Data Availability

This article has no additional data.
